# Efficacy, acceptability and tolerability of the new oral phosphate binder Lenziaren® in healthy cats fed a standard diet

**DOI:** 10.1186/s12917-014-0258-8

**Published:** 2014-10-28

**Authors:** Jonathan N King, Heidi L Erasmus, Peet C Delport, Ina CJ Bester, Wolfgang Seewald

**Affiliations:** Novartis Animal Health Inc, Clinical Development, CH-4002 Basel, Switzerland; Clinvet International Ltd, Bloemfontein, Republic of South Africa

**Keywords:** Cat, Lenziaren, Phosphate, Phosphate binder, SBR759

## Abstract

**Background:**

The efficacy, acceptability and tolerability of the new oral phosphate binder Lenziaren® (SBR759) were evaluated in a randomized parallel-group design study in 36 healthy cats (n = 6 per group). Five groups were fed once daily with a commercial diet containing 0.2% phosphorus (“standard diet”) into which was mixed Lenziaren® at 0.25, 0.5, 1.0 or 2.0 g/day or no treatment (control group) daily for 30 days. A sixth group was fed a commercial diet containing lower amounts (0.12%) of phosphorus (“renal diet”) and no treatment.

**Results:**

When compared to the control group, Lenziaren® produced significant dose-related reductions in urine phosphate concentrations, urine phosphate excretion and fractional urinary phosphate excretion. Significant effects versus the control group were observed at the 0.5, 1.0 and 2.0 g/day dosages. Lenziaren® was well tolerated and was associated with higher food consumption and serum iron concentrations versus the control.

When compared to the control group, the renal diet was associated with significantly lower urine phosphate concentrations and loss of body weight. Lenziaren® had similar effects on urine phosphate concentrations compared to the renal diet, but was not associated with loss of body weight.

**Conclusions:**

Lenziaren® was effective as an oral phosphate binder in cats fed with a standard diet containing 0.2% phosphorus. The acceptability and tolerability were good. Dosages of 0.5-1.0 g/cat per day are recommended for clinical testing in cats fed with a standard diet.

## Background

Disorders of calcium and phosphate metabolism are a frequent consequence of chronic kidney disease (CKD) in cats. In cases of CKD, reduction in the excretory function of the kidney can lead to an increase in plasma phosphate concentrations unless oral intake of phosphate is reduced [1]. Elevated plasma phosphate concentrations correlate with a poor prognosis in cats with CKD [2-5] and therefore treatment guidelines for CKD in cats recommend monitoring of plasma phosphate concentrations and, where appropriate, restriction of oral phosphate intake [6].

Restriction of oral intake of phosphate in cats is achieved most commonly via the feeding of diets containing low amounts of phosphorus (hereafter referred to as “renal diet”). Benefits of renal diets in cats have been reported in experimental models [7], prospective field studies [2,8] and a retrospective field study [9]. However renal diets may not be effective alone in maintaining plasma phosphate concentrations in the recommended target ranges [6] and many renal diets have relatively low palatability in cats [2,10]. Phosphate binders, which reduce the oral absorption of dietary phosphate, are therefore useful agents in the management of hyperphosphataemia [11].

A number of phosphate binders are used in cats including Ipakitine® (calcium carbonate), Lantharenol® or Renalzin® (lanthanum carbonate octahydrate) and various products registered for use in humans containing aluminium or calcium [11,12]. However, none of the available phosphate binders are ideal for use in cats due to low palatability and/or safety issues related to the oral absorption of aluminium, calcium or lanthanum.

Lenziaren® (SBR759) is a new oral phosphate binder which consists of an insoluble complex of iron (III) oxide/hydroxide with starch and sucrose. Lenziaren® does not contain aluminium, calcium or lanthanum, and therefore may have safety advantages over phosphate binders containing those cations. The efficacy of SBR759 in reducing serum phosphate concentrations has been demonstrated in humans undergoing haemodialysis [13,14]. In healthy cats fed with a renal diet, Lenziaren® was effective in reducing plasma and urine phosphate concentrations over the dose range 0.25-1.0 g/cat per day and had good acceptability and tolerability [15]. The efficacy and tolerability of Lenziaren® in cats with CKD and hyperphosphataemia and safety in healthy cats have been reported as abstracts [16,17].

Since not all cats will eat sufficient quantities of renal diets, it is important to determine the efficacy of phosphate binders in cats fed with a “standard” diet. Higher doses of phosphate binders are anticipated to be needed with standard diets due to their higher content of phosphorus compared to renal diets.

The objective of this study was therefore to evaluate the efficacy, dose-effect relation, acceptability and tolerability of Lenziaren® in healthy cats fed a commercial standard diet containing 0.2% phosphorus. The study was a prospective, randomized, masked, parallel-group comparison of six groups. Five groups received four different dosages of Lenziaren® or no treatment (control) plus standard diet. The sixth group was fed with a prescription renal diet containing 0.12% phosphorus.

## Methods

### Study animals and approvals

A total of 36 healthy domestic cross-bred cats (18 female and 18 male) were used. Since one group of cats was fed a renal diet with restricted phosphate for a total of up to 9 weeks, and phosphate is necessary for growth of young animals, the minimum age at study start was 12 months. Each cat was identified via a unique subcutaneous transponder.

The study was conducted at Clinvet International Ltd (Bloemfontein, Republic of South Africa) in compliance with the OECD principles of Good Laboratory Practice, and after approval from the Clinvet Animal Ethics Committee and Novartis Animal Heath Animal Welfare Committee. The study was conducted with reference to the following additional guidelines: VICH guideline GL 43 on target animal safety for pharmaceuticals (EMEA/CVMP/VICH/393388/2006) and The South African National Standard SANS 10386:2008 “The care and use of animals for scientific purposes.” There were no suitable non-animal models and therefore *in vivo* studies were required to quantify the effect of Lenziaren® on phosphate balance in cats. The study did not induce pain or distress in the cats, which were purpose-bred for laboratory studies. Since faeces and urine had to be collected individually from each animal, the cats were housed for the duration of the 9 week study individually in stainless steel cages in an indoor animal unit. Each cage was fitted with a rest bench and scratch block, and cats were interacted with socially at least once per day. The temperature was 20 (±4) °C with a 12:12 hour light:dark cycle.

### Study schedule

The study schedule is shown in Table 1. The cats were acclimatized to the cages and introduced to their diets from day −30. Allocation to the groups (3 females and 3 males per group) was according to randomization through minimization with body weight as the only criterion.

**Table 1:** **Study schedule**

**Study day**	**Action**
−30	Body weight and blood collection for serum phosphate
	Ranking and allocation to groups
	Start of acclimatization to housing and diets
	Start of daily general health observations
−20	Body weight
	Clinical examination
−16 to −12, −9 to −6 and −1	Acceptability test
−14	Blood collection for chemistry and haematology
	72 hour urine collection (baseline) (−14 to −12)
	72 hour faeces collection (baseline) (−14 to −12)
−1	Clinical examination
0	Start daily administration of Lenziaren® to groups A-D (day 0 to +29)
	Specific health observations (including hourly for 4 hours post administration of test item)
0 to +2, +5 to +8, +12 to +14	Acceptability test
+6	72 hour urine collection (+6 to +9)
	72 hour faeces collection (+6 to +9)
+7	Clinical examination
	Body weight
	Blood specimen collection
+13	Clinical examination
	72 hour urine collection (+13 to +16)
	72 hour faeces collection (+13 to +16)
+14	Body weight
	Blood specimen collection
+20	Clinical examination
	72 hour urine collection (+20 to +23)
	72 hour faeces collection (+20 to +23)
+21	Body weight
	Blood specimen collection
+27	Clinical examination
	72 hour urine collection (+27 to +30)
	72 hour faeces collection (+27 to +30)
+28	Body weight
	Blood specimen collection
+29	Final administration of Lenziaren®
+30	End of biological phase of study

Deviations of ±2 days were permitted for some activities for logistical reasons.

General health observations were performed daily, and detailed clinical examinations were made on days −20, −1, 7, 13, 20 and 27. Lenziaren® may cause darkening of the faeces due to its iron content, with no known adverse consequences. Occurrences of darkening of the faeces, without other detected adverse effects, were therefore recorded but were not defined as adverse events. Body weight was recorded on study days −30, −20, 7, 14, 21 and 28. Food was removed approximately 8–10 hours before weighing.

### Test item

The cats received either 0.25, 0.5, 1.0 or 2.0 g/day Lenziaren® (Novartis Animal Health Inc, Basel, Switzerland) or no treatment. The Lenziaren® was weighed and sealed in plastic sachets for each cat before the day of administration, and was then mixed on days 0 to 29 with the entire daily food ration which was offered once per day in the morning. Lenziaren® is a powder and was sprinkled onto the food which was stirred gently for at least 30 seconds to allow the powder to stick to food in a homogeneous manner.

No placebo was used in this study. Since the iron content of Lenziaren® adds colour and is responsible for its efficacy, no suitable placebo was available. Since diets in groups A-E were different versus group F, assessments of the important endpoints of the study were kept masked by use of different personnel. Persons not otherwise involved in the study prepared the diet and test items in sachets and assigned codes (A-F) to the study groups, and were not blinded. The people who administered the test items to the cats, and assessed the feed consumption and acceptability of the test items, could only identify the groups by their codes. The investigators responsible for clinical examinations were fully blinded since they had no access to the study codes and were kept out of sight of the food. At no stage in the study was it necessary to reveal the blinding code to blinded personnel. The blinding code was revealed only after the data base had been locked.

### Diets

The cats were fed one of two wet diets. The standard diet consisted of Hill's Science Plan Adult 1-6 Optimal Care Chicken (Hill's Pet Nutrition Inc, Topeka, KS, USA). Information from the manufacturer lists that the diet contains 10.4% crude protein, 6.7% crude oils and fats, 0.20% calcium and 0.18% phosphorus.

The renal diet consisted of Hill’s Prescription® Diet k/d® Feline Renal Health with Chicken (14%). Information from the manufacturer lists that the diet contains 7.6% crude protein, 7.0% crude oils and fats, 0.17% calcium and 0.1% phosphorus.

Samples of each batch of food were analyzed to confirm the phosphorus content; mean (range) values were 0.2 (0.18-0.22)% for the standard diet (n = 32 batches) and 0.12 (0.09-0.14)% for the renal diet (n = 9 batches).

The cats were acclimatized to their cages and introduced to their diets (standard diet in groups A-E, renal diet in group F) from day −30. The daily intake of each cat was recorded during the last week of acclimatization and this amount of food was then offered to each cat daily on days 0 to 29.

Water (*ad libitum*) and feed were offered in separate stainless steel bowls.

### Assessments

On each day from 0 to 29, the amount of food offered and not eaten was weighed and the percentage of food consumed calculated as follows:

$$ \mathrm{Percentage}\ \mathrm{of}\ \mathrm{food}\ \mathrm{consumed}\ \left(\%\right)=\frac{\mathrm{Amount}\ \mathrm{of}\ \mathrm{food}\ \mathrm{consumed}}{\mathrm{Amount}\ \mathrm{of}\ \mathrm{food}\ \mathrm{of}\mathrm{fered}}\times 100 $$

The dose of the test item consumed was calculated by multiplying the percentage of food consumed (as calculated above) by the nominal dose administered (Table 2). Since the test item was mixed homogeneously with the food, the percentage of test item consumed was considered equal to the percentage food consumed. Food not eaten 23 hours after offer was removed and considered as not eaten. In the event that a cat did not eat all of its medicated food, additional unmedicated food was not offered.

**Table 2:** **Summary of data - part I**

**Group**	**E**	**A**	**B**	**C**	**D**	**F**
	**Standard diet - control**	**Standard diet +0.25 g/day Lenziaren®**	**Standard diet +0.5 g/day Lenziaren®**	**Standard diet +1.0 g/day Lenziaren®**	**Standard diet +2.0 g/day Lenziaren®**	**Renal diet**
**Body weight (kg)**						
Day −30	3.617 (0.948)	3.445 (0.630)	3.523 (0.880)	3.342 (0.886)	3.413 (0.667)	3.373 (0.536)
Day −20	3.452 (0.894)	3.293 (0.654	3.405 (0.864	3.308 (0.740)	3.355 (0.700)	3.212 (0.490)
Day 28	3.528 (1.003)	3.448 (0.627)	3.500 (1.020)	3.450 (0.745)	3.445 (0.648)	2.953 (0.376)
CFB Day 28 from Day −30	−0.088 (0.228)	0.003 (0.276)	−0.023 (0.225)	0.108 (0.475)	0.032 (0.283)	−0.420 (0.172)
Mean Days 7-28	3.532 (1.012)	3.430 (0.608)	3.462 (0.990)	3.436 (0.738)	3.465 (0.626)	2.965 (0.399)
CFB Mean Days 7–28 from Day −30	−0.085 (0.191)	−0.015 (0.248)	−0.061 (0.186)	0.095 (0.430)	0.051 (0.266)	−0.409 (0.157)
**Food consumed (g/day)**						
Day −14	183.6 (53.9)	200.5 (25.3)	171.3 (57.4)	197.7 (45.5)	220.4 (65.0)	NA
Day 28	174.8 (47.7)	200.4 (25.2)	190.2 (53.4)	202.2 (42.8)	189.5 (51.8)	163.6 (39.0)
CFB Day 28 from Day −14	−8.8 (12.5)	−0.1 (0.2)	18.9 (19.5)	4.5 (21.9)	−30.9 (36.9)	NA
Mean Days 7-28	172.7 (47.0)	198.2 (21.4)	181.9 (46.9)	205.0 (45.7)	198.1 (49.4)	160.2 (41.6)
CFB Mean Days 7–28 from Day −14	−10.9 (16.6)	−2.3 (5.6)	10.6 (17.1)	7.3 (24.2)	−22.3 (34.4)	NA
**Serum phosphate concentration (mmol/L)**						
Day −30	1.64 (0.22)	1.68 (0.36)	1.56 (0.41)	1.66 (0.28)	1.64 (0.42)	1.76 (0.35)
Day 28	1.81 (0.26)	1.71 (0.23)	1.70 (0.15)	1.78 (0.23)	1.68 (0.40)	1.60 (0.10)
CFB Day 28 from Day −30	0.17 (0.27)	0.03 (0.39)	0.15 (0.33)	0.12 (0.25)	0.04 (0.46)	−0.16 (0.27)
Mean Days 7-28	1.71 (0.26)	1.70 (0.15)	1.68 (0.13)	1.77 (0.22)	1.70 (0.36)	1.61 (0.14)
CFB Mean Days 7–28 from Day −30	0.07 (0.29)	0.02 (0.27)	0.12 (0.33)	0.11 (0.21)	0.06 (0.44)	−0.15 (0.22)
**Urine phosphate concentration (mmol/L)**						
Day −14	67.5 (12.4)	71.8 (36.4)	77.7 (26.4)	78.8 (26.4)	68.7 (16.6)	NA
Day 28	85.2 (22.8)	78.7 (19.8)	70.1 (14.6)	59.4 (16.3)	63.0 (12.5)	61.7 (12.2)
CFB Day 28 from Day −14	17.7 (17.6)	6.9 (25.4)	−7.6 (34.6)	−19.5 (22.7)	−5.7 (15.0)	NA
Mean Days 7-28	86.3 (23.9)	82.3 (23.5)	77.4 (14.1)	71.8 (15.4)	59.1 (16.6)	65.8 (17.0)
CFB Mean Days 7–28 from Day −14	18.8 (15.9)	10.5 (18.5)	−0.3 (27.1)	−7.0 (16.3)	−9.6 (20.8)	NA

Data are mean (SD), *CFB* = change from baseline.

*NA* = not applicable.

The acceptability of the food, with or without the test items, was assessed from the time for each cat to eat all the food offered during 10 of the last 16 days of acclimatization (study days −16 to −1) and during 10 of the first 15 days of the study (days 0 to 14). The time during which the food was consumed was recorded, to the nearest one hour, hourly for the first six hours, and then at approximately 23 hours. If there was still food remaining at 23 hours, the time during which the food was consumed was assumed to be 24 hours.

Blood specimens were collected under sedation (xylazine and atipamezole) on days −30, −14, 7, 14, 21 and 28 without coagulant for serum chemistry and into EDTA for haematology. Food was removed approximately 8–10 hours prior to blood collection and weighing. Serum chemistry variables included alanine aminotransferase (ALT), alkaline phosphatase (ALP), total calcium, creatinine, iron, phosphorus (serum inorganic phosphate) and urea. Haematology variables included red cell count, haematocrit, haemoglobin, total and differential white cell count, platelet count, prothrombin time and activated partial thromboplastin time.

Specimens of faeces and urine were collected separately on days −14 to −12, 6 to 9, 13 to 16, 20 to 23, and 27 to 30. For each collection session, litter trays containing Katkor® (Rein Vet Products, The Netherlands [18]) were placed in the cages during the afternoon and removed in the morning 3 to 4 days later. This ensured the collection of urine (and faeces) during at least 72 hours.

Faeces were collected at least twice a day during each 72 hour collection session. Pieces of Katkor® that had adhered to faeces were removed. Faeces were then pooled, frozen and weighed before being freeze dried and milled to create a fine powder to ensure homogeneity. The faecal samples were then analyzed in one run for phosphorus content using method KJ4 (Association of Official Analytical Chemists Inc, 1984, Official Methods of Analysis, 14th Ed).

Urine specimens (≥ 500 μL) were collected at least twice a day from the trays containing Katkor®. Alternative methods (e.g. catheter, cystocentesis or manual expulsion) were not needed. Because urine concentration in cats is not constant, a 72 hour pooled specimen was collected at each collection session. Urine from each 72 hour collection session in each cat was pooled in two separate containers, weighed and refrigerated. One container contained 6 N hydrochloric acid to create a pH of ≤ 3-3.5 in order to stabilize the phosphorus and the other container contained no additives. The variables measured were creatinine, phosphate and specific gravity.

Faecal phosphorus excretion was calculated by multiplying the weight of the faeces by the concentration of phosphorus in the faeces.

Urinary phosphate excretion was calculated by multiplying the urine volume (mL) by the concentration of phosphate in the urine. In all samples the urine was weighed (g) and the volume calculated from the measured urine specific gravity. The concentration of phosphate in urine was reported as mmol/L, and was transformed to concentration of phosphorus (g/L) by multiplying by a factor of 0.03097.

The total phosphorus excretion was calculated by adding the urinary phosphorus values to the faecal phosphorus values.

The phosphorus balance was calculated by:

$$ \mathrm{Phosphorus}\ \mathrm{balance}\ \left[\mathrm{g}\right] = \mathrm{Phosphorus}\ \mathrm{intake}\ \hbox{--}\ \mathrm{Total}\ \mathrm{phosphorus}\ \mathrm{excretion}\ \left(\mathrm{faeces}\ \mathrm{and}\ \mathrm{urine}\right) $$

The fractional urinary excretion was calculated by:

$$ \mathrm{Fractional}\ \mathrm{urinary}\ \mathrm{phosphate}\ \mathrm{excretion}\ \left[\%\right]=\frac{\mathrm{Phosphate}\ \mathrm{in}\ \mathrm{Urine}\times \mathrm{Creatinine}\ \mathrm{in}\ \mathrm{Serum}}{\mathrm{Phosphate}\ \mathrm{in}\ \mathrm{Serum}\times \mathrm{Creatinine}\ \mathrm{in}\ \mathrm{Urine}} \times 100 $$

A correction factor of 1/1000 was applied to correct for differences in units.

### Concomitant treatments

Medications known or suspected to be at risk of interacting with the test item or the evaluations used in the study, or with known adverse effects in cats, were not permitted during the study. These included anti-hypertensive agents (including calcium channel blockers and angiotensin-converting enzyme inhibitors), corticosteroids, diuretics, nephrotoxic agents, non-steroidal anti-inflammatory drugs, other phosphate binding agents or any product containing phosphates or phosphorus.

### Statistics

All analyses and calculations were performed using SAS® Version 9.2 (SAS Institute Inc, Cary, NC, USA). Unless stated, data are presented as mean and SD or median and range. Statistical significance was concluded with two-sided P values less than 0.05. No correction was made for multiple analyses (in order not to inflate the type II error).

With the exception of acceptability scores, all efficacy variables were analyzed using repeated measures analysis of covariance (RMANCOVA). An auto-regressive (1) correlation structure between data for the same subject was assumed. Data were log transformed in some cases to improve the fit of the model to a normal distribution. In the primary analysis the effects included in the model were treatment, day, treatment x day interaction, and with or without baseline as covariate (see Results section). In a secondary analysis, percentage of food consumed was included as an additional covariate. In the event of a significant overall treatment effect in the RMANCOVA analysis, pair-wise group comparisons were obtained from the RMANCOVA model with no correction for multiple comparisons. A trend analysis of dose of SBR759 versus each variable was included.

We attempted to evaluate the dose–response relation of Lenziaren® on serum and urine phosphate concentrations using data from groups A-E. However the standard Hill model (sigmoidal curve) could not be applied as either convergence of the model was not achieved or the slope estimate was not significantly different from zero.

Acceptability was assessed as the time for the cat to consume their daily ration. Groups were compared using the Kruskal-Wallis test with post-hoc comparisons using the Mann–Whitney test.

Clinical chemistry, haematology and urine variables were evaluated using RMANCOVA (or analysis of covariance (ANCOVA) if only two time points were available) with baseline as covariate and day, treatment and treatment x day interaction as effects.

## Results

### Cats

The experimental phase of the study was conducted from March to May 2013. The cats were aged 12–84 months at study day −30, and weighed 2.42-3.61 kg (females) and 3.03-4.98 kg (males). With the exception of some minor clinical signs which were judged to be unrelated to the test item, e.g. conjunctivitis, loose faeces or otitis externa, all cats remained in good health throughout the study. No adverse events judged related to Lenziaren® were observed. As defined in the protocol, darkening of the faeces is an expected effect of Lenziaren® due its iron content and has no adverse consequences, and therefore was not reported as an adverse event.

### Test item

The dose of Lenziaren® consumed was estimated from the percentage amount of food consumed, which for days 1–28 was 94.5% for the control group E, 99.0%, 94.2%, 98.2% and 90.6% respectively for Lenziaren® groups A-D, and 96.8% for the renal diet group (F). The estimated daily doses of Lenziaren® were therefore respectively 0.244, 0.465, 0.986 and 1.83 g/day versus the nominal doses of 0.25, 0.5, 1.0 and 2.0 g/day.

### Efficacy endpoints

Summary results for the efficacy endpoints are shown in Tables 2 and 3. Results of the RMANCOVA analyses are reported in Tables 4 and 5. In the primary statistical analysis, the effects tested were treatment, day and treatment x day interaction, with or without baseline as covariate. It was planned in the protocol to include baseline as a covariate for all variables in the analyses. However, group F (fed with renal diet) lost body weight during the study, including during the day −30 to day −1 acclimatization period. Baseline values at days −30 and −20 for body weight and day −30 for serum phosphate concentration were available for all groups. For all other variables, the first “baseline” value was obtained on day −14. Therefore it was decided to perform the following RMANCOVA analyses:

1. Groups A-E (Lenziaren® and negative control) were compared using the baseline values at day −30 for body weight and serum phosphate concentration, and day −14 for all other variables, since groups A-E were managed the same before day 0.
2. Groups A-F were compared for two variables, body weight and serum phosphate concentration, using the available baseline value at day −30, since all groups were managed the same before day −29.
3. Groups A-F were compared for all variables except body weight and serum phosphate concentration without baseline in the model, since the group F was not managed the same as groups A-E between days −29 and −1.

**Table 3:** **Summary of data - part II**

**Group**	**E**	**A**	**B**	**C**	**D**	**F**
	**Standard diet - control**	**Standard diet +0.25 g/day Lenziaren®**	**Standard diet +0.5 g/day Lenziaren®**	**Standard diet +1.0 g/day Lenziaren®**	**Standard diet +2.0 g/day Lenziaren®**	**Renal diet**
**Phosphorus consumed (g/3 days)**						
Day −14	1.102 (0.323)	1.203 (0.152)	1.028 (0.344)	1.186 (0.273)	1.322 (0.390)	NA
Day 28	1.049 (0.286)	1.202 (0.151)	1.141 (0.320)	1.213 (0.257)	1.137 (0.311)	0.567 (0.135)
CFB Day 28 from Day −14	−0.053 (0.075)	−0.001 (0.001)	0.113 (0.117)	0.027 (0.132)	−0.185 (0.222)	NA
Mean Days 7-28	1.036 (0.282)	1.189 (0.128)	1.091 (0.281)	1.230 (0.274)	1.188 (0.296)	0.555 (0.144)
CFB Mean Days 7–28 from Day −14	−0.066 (0.100)	−0.014 (0.034)	0.064 (0.103)	0.044 (0.145)	−0.134 (0.207)	NA
**Phosphorus excreted in faeces (g/3 days)**						
Day −14	0.708 (0.475)	0.778 (0.260)	0.694 (0.415)	0.798 (0.318)	0.735 (0.128)	NA
Day 28	0.810 (0.197)	0.960 (0.222)	0.888 (0.447)	0.877 (0.304)	0.730 (0.245)	0.433 (0.085)
CFB Day 28 from Day −14	0.102 (0.390)	0.182 (0.251)	0.194 (0.375)	0.080 (0.114)	−0.005 (0.165)	NA
Mean Days 7-28	0.716 (0.153)	0.906 (0.205)	0.819 (0.256)	0.901 (0.268)	0.809 (0.287)	0.338 (0.075)
CFB Mean Days 7–28 from Day −14	0.008 (0.360)	0.127 (0.165)	0.125 (0.245)	0.103 (0.131)	0.074 (0.234)	NA
**Phosphorus excreted in urine (g/3 days)**						
Day −14	0.257 (0.083)	0.179 (0.134)	0.295 (0.114)	0.159 (0.060)	0.240 (0.149)	NA
Day 28	0.390 (0.190)	0.420 (0.120)	0.321 (0.117)	0.320 (0.148)	0.166 (0.118)	0.229 (0.091)
CFB Day 28 from Day −14	0.068 (0.132)	0.163 (0.122)	0.040 (0.055)	0.167 (0.142)	−0.020 (0.086)	NA
Mean Days 7-28	0.353 (0.111)	0.383 (0.105)	0.320 (0.115)	0.304 (0.109)	0.204 (0.147)	0.189 (0.049))
CFB Mean Days 7–28 from Day −14	0.096 (0.089)	0.204 (0.081)	0.025 (0.058)	0.144 (0.124)	−0.037 (0.098)	NA
**Total phosphorus excreted (g/3 days)**						
Day −14	0.965 (0.549)	0.958 (0.327)	0.989 (0.522)	0.957 (0.312)	0.976 (0.204)	NA
Day 28	1.201 (0.331)	1.380 (0.230)	1.209 (0.490)	1.197 (0.439)	0.896 (0.328)	0.661 (0.166)
CFB Day 28 from Day −14	−0.011 (0.437)	0.179 (0.188)	0.145 (0.301)	0.243 (0.235)	0.123 (0.408)	NA
Mean Days 7-28	1.068 (0.247)	1.289 (0.277)	1.139 (0.354)	1.205 (0.372)	1.013 (0.405)	0.526 (0.118)
CFB Mean Days 7–28 from Day −14	0.103 (0.370)	0.331 (0.197)	0.150 (0.245)	0.247 (0.136)	0.037 (0.261)	NA
**Phosphorus balance (g/3 days)**						
Day −14	0.137 (0.283)	0.245 (0.296)	0.039 (0.245)	0.229 (0.200)	0.347 (0.254)	NA
Day 28	−0.152 (0.208)	−0.178 (0.282)	−0.068 (0.348)	0.016 (0.327)	0.241 (0.192)	−0.094 (0.052)
CFB Day 28 from Day −14	−0.288 (0.300)	−0.423 (0.262)	−0.106 (0.263)	−0.213 (0.240)	−0.106 (0.206)	NA
Mean Days 7-28	−0.032 (0.197)	−0.100 (0.262)	−0.048 (0.171)	0.025 (0.220)	0.175 (0.177)	0.029 (0.047)
CFB Mean Days 7–28 from Day −14	−0.169 (0.369)	−0.345 (0.187)	−0.087 (0.165)	−0.204 (0.113)	−0.171 (0.341)	NA
**Fractional urinary phosphate excretion (%)**						
Day −14	17.96 (4.47)	16.57 (3.36)	20.75 (4.63)	19.64 (5.30)	17.60 (4.71)	NA
Day 28	18.60 (7.21)	17.91 (5.70)	18.44 (5.71)	15.17 (2.25)	11.37 (3.71)	16.97 (2.41)
CFB Day 28 from Day −14	0.63 (4.02)	1.34 (4.15)	−2.32 (6.01)	−4.47 (5.22)	−6.23 (1.49)	NA
Mean Days 7-28	20.39 (6.68)	19.18 (3.43)	17.90 (4.34)	17.25 (2.23)	12.93 (5.09)	15.27 (3.26)
CFB Mean Days 7–28 from Day −14	2.42 (3.36)	2.61 (2.25)	−2.85 (4.43)	−2.39 (3.58)	−4.67 (2.16)	NA

Data are mean (SD), *CFB* = change from baseline.

*NA* = not applicable.

**Table 4:** **Summary of RMANCOVA analyses for control and Lenziaren® groups only (groups A-E)**

**Variable**	**Transformation**	**Normality P value**	**P values for model effects in RMANCOVA**					
			**Treatment**	Baseline*	**Day**	**Treatment x day interaction**	**P < 0.05 in post hoc comparisons**	Dose trend**
**Body weight**	Log	0.1698	0.9223	**< 0.0001**	0.4523	0.2702	None significant	0.31
**Food consumed**	Log	**< 0.0001**	**0.0434**	**< 0.0001**	0.9959	0.4724	**B & C > D & E**	0.91
**Serum phosphate concentration**	Log	0.5181	0.1501	**< 0.0001**	0.4910	0.4898	**D > E**	**0.020 inc**
**Urine phosphate concentration**	None	0.4201	**0.0076**	**0.0005**	**0.0428**	**0.0455**	**C < E**	**0.0003 dec**
							**D < A, B & E**	
**Phosphorus consumed**	None	**< 0.0001**	**0.0266**	**< 0.0001**	0.9904	0.4527	**B & C > D & E**	0.9299
**Phosphorus excreted in faeces**	Log	0.6280	0.4867	**< 0.0001**	**< 0.0001**	0.6309	None significant	0.4854
**Phosphorus excreted in urine**	Log	0.6590	**0.0040**	**0.0001**	**0.0087**	0.1695	**B < A**	**0.0064 dec**
							**D < A, C & E**	
**Total phosphorus excreted**	Log	0.3264	0.1518	**< 0.0001**	**< 0.0001**	0.3878	**A < D**	0.0938
**Phosphorus balance**	Log	**0.0242**	0.2554	**0.0184**	**< 0.0001**	0.8813	**A < D**	0.0878
**Fractional urinary phosphate excretion**	None	0.6858	**0.0001**	**< 0.0001**	**0.0222**	0.8098	**B & C < A & E**	**< 0.0001 dec**
							**D < A, B, C & E**	

*The baseline used in the model was day −30 for body weight and serum phosphate concentration, and day −14 for all other variables.

**dec = decrease, inc = increase.

Values in bold indicate statistical significance (P < 0.05).

**Table 5:** **Summary of RMANCOVA analyses for all groups (A-F)**

**Variable**	**Transformation exponent**	**Normality P value**	**P values for model effects in RMANCOVA**				
			**Treatment**	Baseline*	**Day**	**Treatment x day interaction**	**P < 0.05 in post hoc comparisons**
**Body weight**	Log	**0.0009**	**0.0296**	**< 0.0001**	0.8576	0.1674	**A, B, C, D & E > F**
**Food consumed**	Log	**0.0049**	0.3882		0.9961	0.7012	None significant
**Serum phosphate concentration**	Log	**0.0266**	0.6387	**0.0030**	0.5919	0.3622	None significant
**Urine phosphate concentration**	None	0.5206	**0.0337**		**0.0023**	0.0714	**D < A, B & E F < E**
**Phosphorus consumed**	None	**0.0259**	**< 0.0001**		0.9806	0.7083	**F < A, B, C, D & E**
**Phosphorus excreted in faeces**	Log	0.9238	**0.0004**		**< 0.0001**	0.1576	**F < A, B, C, D & E**
**Phosphorus excreted in urine**	Log	**0.0445**	**0.0196**		**0.0123**	0.1278	**D < A & E F < A, B & E**
**Total phosphorus excreted**	Log	0.3241	**0.0021**		**< 0.0001**	**0.0470**	**F < A, B, C, D & E**
**Phosphorus balance**	Log	**0.0024**	0.1410		**< 0.0001**	0.2730	**D < A, B & E**
**Fractional urinary phosphate excretion**	None	**0.0187**	**0.0456**		**0.0241**	0.3375	**D < A, B, C & E**

*The baseline in the model for variables body weight and serum phosphate concentration was day −30. For all other variables, no baseline was included in the model.

Values in bold indicate statistical significance (P < 0.05).

In a secondary analysis, percentage of food consumed was included as an additional covariate and was significant (*P* < 0.05) for many variables (serum and urine phosphate concentrations, phosphorus consumed, phosphorus excreted in faeces and in total, and phosphorus balance). However there were no relevant differences in results (including overall treatment effect, dose-trend test or post-hoc group comparisons) for any variable with and without percentage food consumed included as a covariate. Therefore results of the secondary analyses, with food consumption included as a covariate, are not shown in this paper.

In one half of the RMANCOVA analyses model data did not fit a normal distribution, in spite of the use of a log transformation in 70% of the analyses (Tables 4 and 5).

### Body weight

**Figure 1:**
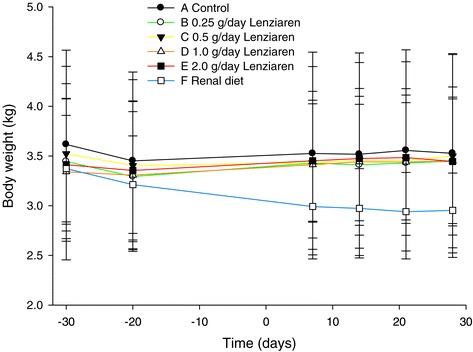
**Mean and SD body weight.** Renal diet (group F) or standard diet (groups A-E) were fed from day −30 to 29. Lenziaren® was administered to groups A, B, C and D on days 0 to 29.

There were no significant differences in body weights between groups A-E and no dose trend for Lenziaren® (Figure 1, Table 4). When group F was included in the analyses (Table 5), there was a significant treatment (*P* = 0.0296) effect and in the post-hoc analyses the body weight in group F was significantly lower than groups A-E. The change from baseline analysis (day −30 to day 30) showed a mean (%) 0.03 kg (0.9%) weight gain with the Lenziaren® groups A-D combined, 0.42 kg (12.4%) weight loss in group F, and a 0.088 kg (2.4%) weight loss in the control group E (Figure 1, Table 2). Cats in group F lost most body weight from days −30 to 7 with a lesser decline from day 7 to day 28 (Figure 1).

### Food consumed

For the day 0 to 30 period, the mean food consumption was respectively 14.7, 5.3, 18.7 and 14.7% higher in the 0.25, 0.5, 1.0 and 2.0 g/day Lenziaren® groups A-D compared to the control group E (Table 2). There was a significant treatment effect (*P* = 0.043) with groups B and C having significantly higher food consumption than groups D and E (Table 4).

When group F was included in the model, there was no significant treatment effect (*P* = 0.39) although the mean food consumption on days 7–28 in group F (160.2 g/day) was 7.3% lower than the control group E (172.7 g/day). Mean food consumption in group F increased progressively during the study: mean daily food consumption in group F was 150.1, 156.1, 159.6, 161.3 and 163.6 g/day on days −14, 7, 14, 21 and 28 respectively (Table 5).

### Acceptability

The acceptability of the food and Lenziaren® was assessed from the amount of uneaten food remaining at the end of every hour for six hours for 10 days before (days −16 to −1) and for 10 days after (days 0 to 14) administering the test item. There were no significant differences either for the all-group comparisons using the Kruskal-Wallis test or for paired comparisons between groups using the Mann–Whitney test (data not shown). Therefore it was concluded that Lenziaren® had no significant effect on acceptability of the standard diet. The number of cats which did not consume all of their food within 23 hours in groups A-F was respectively 1, 3, 5, 5, 3 and 4 over the day 6–8 period and 3, 2, 3, 4, 2 and 3 over the day 26 to 28 period.

### Serum phosphate concentration

There was no significant treatment effect on serum phosphate concentrations for the group A-E or A-F analyses (Tables 4 and 5). However in the group A-E analysis, there was a significant (*P* = 0.02) dose trend test with higher serum phosphate concentrations with increasing dose of Lenziaren®.

### Urine phosphate concentration

In the group A-E analysis, there were highly significant treatment effect (*P* = 0.0076) and dose trend (*P* = 0.0003) tests, with lower urine phosphate concentrations with increasing dose of Lenziaren® (Table 4). In the post-hoc analyses, urine phosphate concentrations were significantly lower in groups C and D (1.0 and 2.0 g/day Lenziaren®) compared to the control, and also in D (2.0 g/day Lenziaren®) versus groups A and B.

In the group A-F analysis, there was a significant overall treatment effect (*P* = 0.0337) with significantly lower concentrations in groups D (2.0 g/day Lenziaren®) and F (renal diet) compared to the control (E) group (Table 5).

Compared to the control group (E), mean urine phosphate concentrations at day 30 were respectively 7.6, 17.7, 30.3, 26.1 and 27.6% lower in groups A, B, C, D and F (Table 2).

### Phosphorus consumed

There was a significant treatment effect in both the group A-E and A-F analyses. In the post-hoc analyses for the group A-E analysis, the phosphorus consumption was higher in groups B and C (0.5 and 1.0 g/day Lenziaren®) compared to groups D and E, reflecting the respective higher food consumption (Table 4).

In the post-hoc analysis for group A-F, the phosphorus consumption was significantly lower in group F (renal diet) compared to all other groups (A-E) (Table 5). This can be explained by a combination of lower dietary phosphate content (0.12% in group F versus 0.2% in groups A-E) plus the lower food consumption in group F.

### Phosphorus excreted in faeces

In the group A-E analysis, there was no significant treatment effect or dose-trend test (Table 4). In the group A-F analysis, there was a significant treatment effect with the amount of phosphorus excreted in the faeces significantly lower in group F (renal diet) compared to all other groups (A-E) (Table 5). This result correlates with the lower dietary content of phosphorus and food consumption in group F.

### Phosphorus excreted in urine

In the group A-E analysis, there was a significant treatment effect (*P* = 0.0040) and dose-trend test (*P* = 0.0064) with a lower amount of phosphorus excreted in the urine with higher dose of Lenziaren® (Table 4). In the post-hoc tests, the amount excreted was lower in group D (2.0 g/day Lenziaren®) versus A, C and E, and in group B (0.5 g/day Lenziaren®) versus A.

In the group A-F analysis, there was a significant treatment effect (*P* = 0.0196) and in the post-hoc comparisons group F (renal diet) had lower values than groups A, B and E (Table 5).

### Total phosphorus excreted

In the group A-E analysis, there was no treatment effect or dose trend test in the total phosphorus excreted (Table 4).

In the group A-F analysis, there was a significant (*P* = 0.0021) treatment effect, and in the post-hoc analysis values were lower with group F (renal diet) versus all other groups (A-E) (Table 5).

### Phosphorus balance

There was no significant treatment effect in both the group A-E and group A-F analyses (Tables 4 and 5).

### Fractional urinary excretion

In the group A-E analysis, there was a highly significant treatment effect (*P* = 0.0001) and dose-trend test (*P* < 0.0001) with lower fractional excretion with higher dose of Lenziaren® (Table 4). In the post-hoc comparisons, the excretion was significantly lower in groups B and C (0.5 and 1.0 g/day Lenziaren®) versus A and E, and in group D (2.0 g/day Lenziaren®) versus A, B, C and E.

At the day 30 time point, the fractional excretion was 3.7, 0.9, 18.4 and 38.9% lower in groups A, B, C and D respectively compared to the control group E (Table 3).

In the group A-F analysis, there was a significant treatment effect (*P* = 0.046) but no significant effect of group F (renal diet) in the post-hoc analysis (Table 5). The fractional urinary excretion in group F was 8.8% lower than the control group.

### Clinical chemistry, haematology and urine variables

In the comparisons of groups A-E, there were significant dose trends for ALP (*P* = 0.012, decrease), ALT (*P* = 0.023, decrease), creatinine (*P* = 0.0070, increase) and iron (*P* = 0.0049, increase). In the post-hoc comparisons, groups C and D (1.0 and 2.0 g/day) were significantly different from E (control) for ALP, creatinine and iron. Group D was significantly different from E for ALP.

Mean (SD) iron concentrations (μmol/L) from day 0 to 28 were 16.0 (2.9) in group A, 22.0 (6.6) in group B, 21.6 (5.9) in group C, 20.4 (5.2) in group D, 16.2 (7.0) in group E and 21.0 (4.7) in group F. These values represent change from baseline of (μmol/L) 2.0 (1.7), 3.2 (3.8), 5.1 (5.1) and 4.6 (6.8) respectively in the Lenziaren® (A-D), −1.0 (3.3) in the control (E) and −3.8 (5.0) in the renal diet (F) groups.

Amylase, platelet and potassium values were significantly higher in group F (renal diet) compared to group E (control).

## Discussion

The primary objective of this study was to determine the efficacy and dose-effect relation of the new oral phosphate binder Lenziaren® in healthy cats fed with a standard diet (containing ~0.2% phosphorus). This objective was fulfilled by the comparison of four dosages of Lenziaren® (0.25-2.0 g/day) to the control group. The results demonstrate that Lenziaren® effectively bound phosphate present in the diet, as evidenced by the significantly lower urine phosphate concentrations, urine phosphorus excretion and fractional urinary phosphate excretion. These effects occurred in spite of higher food consumption and therefore higher phosphorus intake with Lenziaren® compared to the control group. The efficacy of Lenziaren® was dose-dependent over the tested dosage range of 0.25–2.0 g/day, with significant effects versus the control at 0.5, 1.0 and 2.0 g/day on fractional urinary phosphate excretion, 1.0 and 2.0 g/day on urine phosphate concentration and 2.0 g/day on urine phosphorus excretion. The results indicate that a higher dose of Lenziaren®, approximately two times higher, was required to reduce urine phosphate concentrations by an equivalent amount when administered with standard diet in this study compared to a renal diet in a previous study [15]. This is an expected result due to the higher content of phosphorus in the standard diet (0.2%) compared to the renal diet (0.12%) tested. Therefore we can recommend that Lenziaren® be administered at a starting dose of 0.25 g/day, with an increase up to 1.0 g/day as needed, in cats fed with a renal diet. However a starting dose of 0.5 g/day with increase up to 1.0 g/day as needed may be a better recommendation in cats fed with a standard diet. These predictions require testing in field studies with cats with clinical hyperphosphataemia.

The results, showing that Lenziaren® is an effective oral phosphate binder over the dose range 0.5–2.0 g/day, are consistent with results from previous studies. In healthy cats fed with a renal diet, Lenziaren® significantly reduced serum and urine phosphate concentrations over the dose range 0.25-1.0 g/day [15]. In cats with hyperphosphataemia and CKD, Lenziaren® reduced serum phosphate concentrations over the dose range 0.25-1.0 g/day [17]. The efficacy of SBR759 in human patients with hyperphosphataemia undergoing dialysis has also been reported over the dose range 3.75-22.5 g/day [13].

The tolerability of Lenziaren® in this study was good, as assessed from the lack of reported adverse effects attributed to the test item. Lenziaren® had no detected negative impact on acceptability or intake of the food (standard diet), and in fact food consumption and bodyweight gain were higher with Lenziaren® compared to the control and renal diet groups.

Significantly higher serum creatinine, iron and phosphate concentrations and significantly lower ALP and ALT activities were observed with higher doses of Lenziaren®. An oral phosphate binder would be expected to decrease serum phosphate concentrations, however homeostatic mechanisms in healthy cats could have maintained serum concentrations during the relatively short duration (30 days) of administration of Lenaziaren®. It is not clear why the serum phosphate concentrations showed a positive trend with dose in this study. It is a fact that the Lenziaren® treated cats consumed higher quantities of phosphate as a result of higher food consumption, but the urine phosphate results suggest that absorption of phosphate was effectively decreased by Lenziaren®. Significant reductions in serum phosphate concentrations by Lenziaren®, as one would expect for a phosphate binder, were reported in previous studies in healthy cats fed a renal diet for 28 days [15] and in cats with CKD [17]. The higher serum creatinine concentrations in this study might be related to the maintenance of body weight with Lenziaren® compared to the control group, but we have no explanation for the dose-related lower ALP and ALT activities with Lenziaren®. Similar effects of Lenziaren® on ALP, ALT and creatinine were not observed in a previous study [15].

Although mean serum iron concentrations in the Lenziaren® groups (range 16.0-22.0 μmol/L) remained within the reference range of cats at the study site, concentrations were significantly higher in the Lenziaren® groups compared to the control group, and increased in a dose-dependent manner. This result is logical since Lenziaren® is a complex of iron with starch and sucrose, designed to increase the binding potency to phosphate and to limit systemic absorption of iron. Nevertheless small amounts of iron can be absorbed. There were slight increases from baseline in mean iron concentrations (+2.0 to +5.1 μmol/L) in the cats receiving Lenziaren®, whereas both the control (−1.0 μmol/L) and renal diet (−3.8 μmol/L) groups showed decreases in iron concentrations. One explanation for these findings is that the blood sampling schedule, although it represented only a total of 63–72 mL of blood over a 9 week period, may have led to decreases in iron concentrations in the control and renal diet groups and this effect was prevented by the administration of Lenziaren®. Dose-related increases in plasma iron concentrations were observed also in cats with hyperphosphataemia and CKD dosed with 0.125-1.5 g/day Lenziaren® and fed with renal or standard diet [17 and Novartis data on file]. The magnitude of change from baseline in iron concentrations was similar in the CKD cats (+3.7 μmol/L) as in this study (+2.0 to +5.1 μmol/L). Therefore the release of iron from the Lenziaren® complex and absorption from the gastrointestinal tract was approximately similar in cats with CKD and in healthy animals. In contrast, absorption of iron from the iron-based phosphate binder PA21 was higher in healthy human subjects compared to patients undergoing dialysis [19]. That result was attributed to the effect that oral absorption of iron is reduced by the presence of uraemia (in humans). No changes in plasma iron concentrations were observed in humans with hyperphosphataemia undergoing dialysis receiving SBR759 at 3.75-22.5 g/day with treatment for 4 weeks [13] or at a mean dose of 6.2 g/day for 12 weeks [14].

Higher plasma iron concentrations might be relevant clinically in cats with CKD. Although excessively high plasma iron concentrations are associated with toxicity [20], moderately increased iron concentrations are unlikely to be detrimental since anaemia occurs commonly in cats with CKD and is a risk factor for mortality [5]. Field studies are required to examine further this topic. No adverse effects of absorption of iron with Lenziaren® were detected in this or previous studies in cats [15,17].

A secondary objective of the study was to compare the efficacy and tolerability of a renal diet with the other groups (standard diet with and without Lenziaren®). As compared to the control group (standard diet without Lenziaren®), the renal diet was associated with significant loss of body weight (0.42 kg, 12.4%) during the 60 day study. The feed consumption was 7.3% lower with the renal diet compared to the standard diet, but differences were not statistically significant. Poor compliance of cats to renal diets due to low palatability has been described previously [2,10]. Cats fed with the renal diet had significantly lower urine phosphate concentrations and total amount of phosphorus excreted in the urine, in addition to lower faecal and total excretion of phosphorus, compared with the standard diet. These results can be explained by the effect of the renal diet in reducing total intake of phosphorus. The fractional urinary phosphate excretion was not changed significantly by the renal diet, as expected, indicating that faecal and urinary phosphorus excretions were reduced equally. The amount of phosphorus consumed by the cats receiving the renal diet was 53.6% of the standard diet. This can be accounted for mainly by the lower phosphorus content (60%) of the renal diet with a smaller contribution from the lower food consumption (93%). In a previous study in the same laboratory, growing cats fed the same renal diet for approximately two months showed a small (+0.23 kg) gain in body weight [15] compared to the loss noted in this study. One possible reason for this discrepancy is that the cats in the previous study received a placebo to SBR759 containing caramel to provide a similar colour to Lenziaren® [15]. As noted in the conclusions of that study, it is possible that the caramel increased the acceptability of the renal diet [15]. In spite of the loss of body weight of the cats receiving the renal diet in this study, the cats were judged to be in good health in the clinical examinations and consumed their feed faster than the control group. No significant changes in haematology or serum chemistry variables were observed that could be attributed to the renal diet.

The main limitations of the study are discussed below. First, complete blinding of the study was not possible since two different diets were tested and no suitable placebo was available for the control group. In a previous study in cats, a placebo was used with a similar starch and sucrose complex as SBR759 (without iron) plus caramel to give a similar colour to Lenziaren®. This placebo was no longer available for this study. In addition the placebo was of only limited value as, although its physical appearance was similar, it did not result in darkening of the faeces as occurs with Lenziaren® as a consequence of its iron content. Since complete blinding was not possible, the study was masked by separation of functions so that personnel responsible for clinical examinations did not know the treatment groups. In addition, personnel making the laboratory analyses, which were the most important endpoints, were blinded to the treatment groups.

Second, blood samples from the cats were collected after sedation with xylazine and atipamezole, which might have affected blood variables, appetite or gastrointestinal motility. However any effects should have been the same in all groups.

Third, the choice of baseline in the statistical analyses including group F (renal diet) was challenging due to the unexpected loss of body weight in the renal diet group, which was most pronounced in the acclimatization period. The plan for the study was to acclimatize all cats to their diets prior to day 0. In a previous study in the same laboratory, a 28 day acclimatization period to the renal diet was sufficient and the cats had no significant changes in feed consumption or body weight during the acclimatization period [15]. The unexpected loss of body weight in the renal diet group in the acclimatization period resulted in the baseline values between days −28 and day −1 being unreliable for the renal diet group (F). For two important variables, body weight and serum phosphate concentration, baseline values at day −30 were available and permitted reliable analyses with RMANCOVA including baseline. For all other variables, no baseline was included in the RMANOVA model. In view of these limitations, the results from the renal diet group should be treated as exploratory only and additional studies are recommended. The primary objective of the study was unaffected by this issue, and the Lenziaren® and control group were compared for all variables using RMANCOVA using the baseline values recorded on day −14 or −30. This was valid since the management of those cats was the same prior to day 0.

Fourth, parametric methods (ANCOVA or RMANCOVA) were used since these are more powerful than non-parametric methods especially for repeated measures designs as used in this study. The data were log transformed in many cases, but nevertheless in 50% of analyses the data in the models deviated significantly from a normal distribution. However it was judged that the distributions were satisfactory, and it has been shown that ANOVA models are relatively robust even when the distribution deviates from normality [21]. Therefore we conclude that the statistical analyses were appropriate for the conclusions reached.

Fifth, the results obtained with the diets tested (Hill’s Science Plan Adult 1–6 Optimal Care Chicken for the standard diet, Hill’s Prescription Diet k/d Feline Renal Heath with Chicken for the renal diet) may not necessarily be extrapolated to other diets.

Lastly, the study was conducted in healthy cats and therefore the results cannot be simply extrapolated to clinical cases of hyperphosphataemia in cats. However efficacy of SBR759 over the dose range 0.25-1.0 g/day was reported in an abstract of a field study in cats with hyperphosphataemia secondary to CKD [17].

## Conclusions

In healthy cats fed a standard diet, Lenziaren® at doses of 0.25-2.0 g/cat per day effectively bound oral phosphate as evidenced by a dose-related reduction in urine phosphate concentrations. Lenziaren® was well tolerated and resulted in higher food consumption and dose-related increases in serum iron concentrations compared to the control group.

As compared to the negative control, the renal diet was associated with lower urine phosphate concentrations but also a loss of body weight. Lenziaren® had similar effects on serum and urine phosphate concentrations compared to the renal diet, and was not associated with loss of body weight.

## Abbreviations

ALP: Alkaline phosphatase
ALT: Alanine aminotransferase
EDTA: Ethylenediaminetetraacetic acid
OECD: Organisation for economic co-operation and development
VICH: Veterinary international conference on harmonization
